# Supplementary material for: Molecular Evidence Supports Five Lineages within Chiropotes (Pitheciidae, Platyrrhini)
Source: Genes (Basel). 2023 Jun 21;14(7):1309. doi: 10.3390/genes14071309 (PMC10379672; doi:10.3390/genes14071309)
Supplement: Supplementary file 1 [file genes-14-01309-s001.zip › genes-2375084-supplementary.pdf]

GCAT TACG GCAT genes

Supplementary Materials

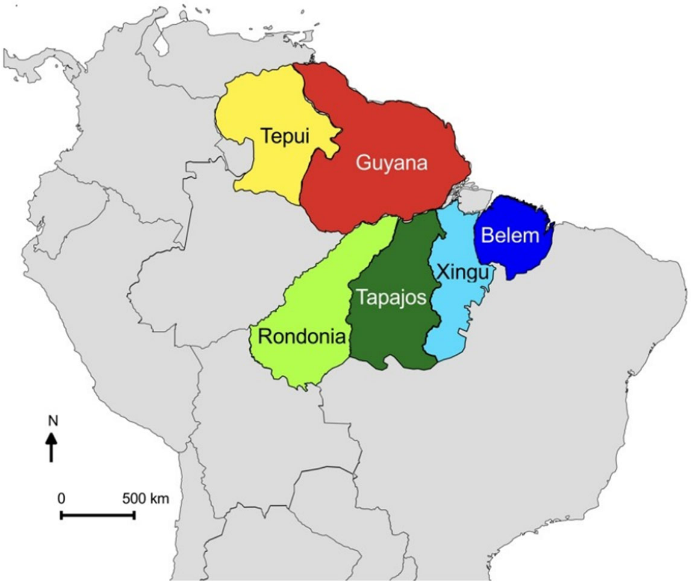

Figure S1. Biogeographic subregions used for the biogeographic analyses: Belem, Xingu, Tapajos, Rondonia, Guyana and Tepui.

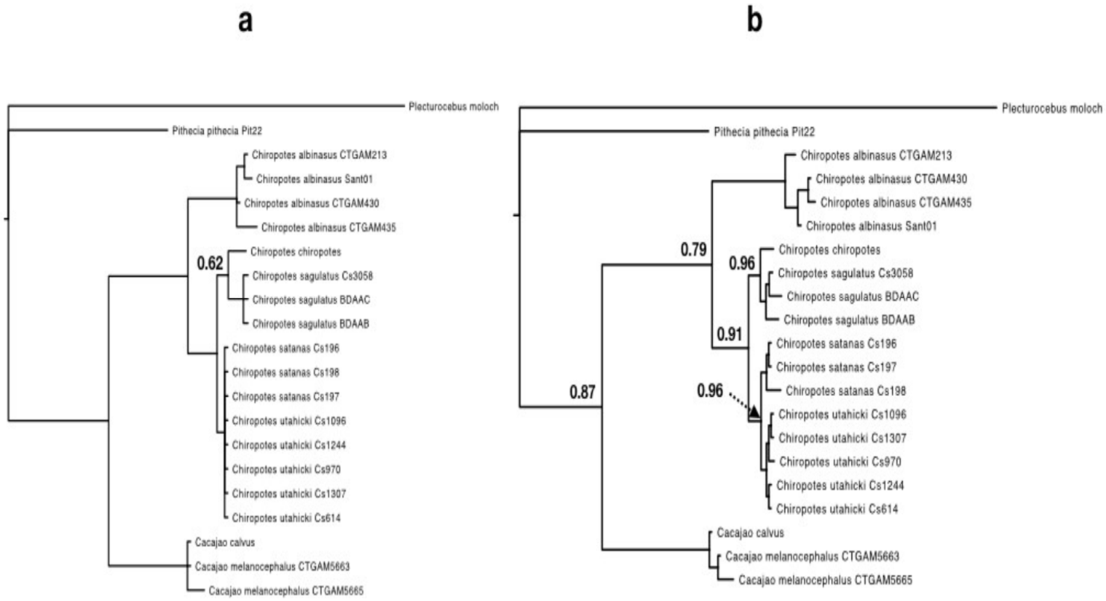

Figure S2. Phylogenetic reconstruction using Bayesian inference (BI) based on six nuclear (a) and two mitochondrial (b) markers. Numbers at nodes indicate the posterior probabilities.

Table S1. Species, molecular markers, and GenBank accession numbers.

Species	Molecular Marker	GenBank Accession Number
Chiropotes chiropotes	ABCA1	HM765295
Chiropotes chiropotes	APP	HM764662
Chiropotes chiropotes	AXIN1	HM764335
Chiropotes chiropotes	CHRNA1	HM763449

Chiropotes chiropotes	DCTN2	HM762785
Chiropotes chiropotes	ERC2	HM762183
Chiropotes chiropotes	COI and Cytb	NC024629
Cacajao calvus	ABCA1	HM765283
Cacajao calvus	APP	HM764655
Cacajao calvus	AXIN1	HM764328
Cacajao calvus	CHRNA1	HM763441
Cacajao calvus	DCTN2	HM762777
Cacajao calvus	ERC2	HM762297
Cacajao calvus	COI and Cytb	NC021967

Table S2. Molecular markers used in the present study and their evolutionary respective models.

Code	Gene	Evolutionary Model	Forward / Reverse
ABCA1 [57]	ATP Binding Cassete subfamily A	K80	CCTCCATCTTTTCAGCTCTACCTAC<br>ACAAGAGCCTGGAGATTGGATAAC
AXIN1[57]	AXIN1	K80	CTCTGCCTTCGCTGTACCGTCTAC<br>GACCCACCTTTCCTAATCCTTGTC
CHRNA1 [58]	Cholinergic receptor, nicotinic alpha 1	K80	GACCATGAAGTCAGACCAGGAG<br>GGAGTATGTGGTCCATCACCAT
DCTN2 [22]	dynactin 2	HKY85	TGGCTCTGGCTCTGTACTCA<br>GAAAACCTGGCCACAGTTGA
ERC2 [57]	ELKS/RAB6 interacting/CAST family member	HKY85+G	AGCTCATCCTCCTCCTGGTTTAG<br>CTCCTTGAGGATCTCCAGCAAC
COI [59]	Cytochrome oxidase subunit 1	HKY85+G	TCAACCAACCACAAAGACATTGGCAC<br>TAGACTTCTGGGTGGCCAAAGAATCA
CYTB [60]	Cytochrome b	HKY85+G	GCACCTACCCACGAAAAGAA<br>ACATTGCCTCTGCAAATTGA

Table S3. T Models, number of parameters, Log-Likelihood (ln L), Dispersal (d), Extinction (e), Founder (j), Geographic Distance (x), Akaike Information Criterion (AIC), corrected Akaike Information Criterion (AICc), and ΔAIC values.

Models	N° Parameters	LnL	D	E	j	X	AIC	AICc	ΔAIC
DEC	2	-13.60	0.049	0.14	0	0	31.20	37.20	10.92
DEC+J	3	-6.90	1.0e-12	1.0e-12	2.39	0	19.79	43.79	17.51
DEC+X	3	-12.01	0.13	0.049	0	-3.55	30.02	54.02	27.34
DEC+J+X	4	-6.30	1.0e-12	1.0e-12	2.61	-1.6	20.60	68.76	42.48
DIVALIKE	2	-8.14	0.0024	1.0e-12	0	0	20.28	26.28	0
DIVALIKE+J	3	-6.42	1.0e-12	4.7e-10	0.37	0	18.38	42.83	16.55
DIVALIKE+X	3	-7.84	0.11	1.0e-12	0	-3.48	21.67	45.67	19.39
DIVALIKE+J+X	4	-5.65	0.020	1.0e-12	0.89	-3.09	19.31	64.39	38.11
BAYAREA	2	-15.04	0.076	0.44	0	0	34.09	40.09	13.80
BAYAREA+J	3	-6.91	1.0e-07	0.0027	1.00	0	19.81	43.81	17.53
BAYAREA+X	3	-14.00	0.35	0.48	0	-2.52	34.00	58.00	31.72
BAYAREA+J+X	4	-6.32	1.0e-12	0.0068	1.00	-1.57	20.63	69.10	42.82
